# Bronchoscopic local instillation of amphotericin B deoxycholate as adjunctive therapy for pulmonary mucormycosis with inadequate response to posaconazole: a retrospective case series

**DOI:** 10.3389/fmed.2026.1840224

**Published:** 2026-08-17

**Authors:** Liyan Qiu, Binyue Liang, Hua Zhou

**Affiliations:** 1 Department of Respiratory and Critical Care Medicine, Quzhou Traditional Chinese Medicine Hospital, Quzhou, China; 2 Department of Respiratory and Critical Care Medicine, The First Affiliated Hospital, Zhejiang University School of Medicine, Hangzhou, China

**Keywords:** airway obstruction, amphotericin B deoxycholate, bronchoscopy, local instillation, posaconazole, pulmonary mucormycosis

## Abstract

**Background:**

Pulmonary mucormycosis is a life-threatening invasive fungal infection, most often affecting immunocompromised patients. Liposomal amphotericin B is the preferred first-line therapy, but amphotericin B formulations may be poorly tolerated. When endobronchial obstruction and tissue necrosis are present, pulmonary lesions may progress despite systemic antifungal therapy.

**Methods:**

We retrospectively reviewed patients with documented pulmonary mucormycosis who received posaconazole-based therapy at the First Affiliated Hospital, Zhejiang University School of Medicine between May 2019 and December 2022. Patients who showed clinical deterioration, radiologic progression, or no meaningful radiologic improvement during posaconazole-based therapy and then underwent bronchoscopic airway clearance followed by local instillation of amphotericin B deoxycholate were included.

**Results:**

Nineteen patients received posaconazole-based antifungal therapy during the study period, and five met the eligibility criteria. Their mean age was 56.2 ± 3.96 years; four had major underlying conditions, including hematologic malignancy or destroyed lung after tuberculosis, and one had a previous history of pulmonary and sinonasal fungal disease. Diagnostic evidence included mNGS, fungal fluorescence staining, bronchoalveolar lavage findings, and/or histopathology. Bronchoscopy identified obstructing mucus plugs, purulent secretions, necrotic material, or segmental narrowing in all patients. Amphotericin B deoxycholate was locally instilled at 7.5–15 mg in 10–15 ml diluent for 2–3 sessions while posaconazole was continued. No treatment-limiting cough, clinically significant oxygen desaturation, hemoptysis, bronchospasm, or new hepatic or renal dysfunction related to the local procedure was documented. Follow-up CT showed partial absorption or shrinkage of the dominant pulmonary lesions in all five patients, and none required surgical resection.

**Conclusions:**

In this small retrospective case series, bronchoscopic airway clearance plus local instillation of amphotericin B deoxycholate appeared feasible and well tolerated as an adjunctive salvage approach for selected patients with pulmonary mucormycosis and inadequate response to posaconazole. Because mechanical clearance and local drug delivery were inseparable in this study, the efficacy of local amphotericin B cannot be established from these data alone.

## Introduction

Mucormycosis is a severe invasive fungal disease caused by fungi of the order Mucorales (1). Common genera include Rhizopus, Mucor, Lichtheimia, and Cunninghamella. Infection may involve the paranasal sinuses, orbit, central nervous system, lungs, gastrointestinal tract, skin, or disseminated sites (1, 2). In mainland China, pulmonary mucormycosis is the most frequently reported clinical form and accounts for about 42% of all published mucormycosis cases (3).

Pulmonary mucormycosis usually develops in patients with impaired host defenses, including those with hematologic malignancy, chemotherapy exposure, transplantation, prolonged corticosteroid use, uncontrolled diabetes, or chronic structural lung disease (4–9). The disease is highly angioinvasive and can lead to thrombosis, tissue necrosis, and infarction. Once necrosis and airway obstruction develop, systemically administered antifungal agents may not reach the lesion effectively. This contributes to poor outcomes and high mortality (4, 10–14).

Current management relies on prompt diagnosis, reversal of pre-disposing factors when possible, systemic antifungal therapy, and surgical intervention in selected patients (10, 12, 15–17). Liposomal amphotericin B remains the preferred first-line antifungal agent. Posaconazole is commonly used as salvage or step-down therapy and as an alternative when amphotericin B is not tolerated (15, 16, 18). However, treatment failure still occurs, particularly when necrotic debris and mucus obstruct the involved bronchi and limit local drug exposure. This clinical problem raises the question of whether direct bronchoscopic delivery of amphotericin B to the lesion may improve local control while avoiding additional systemic toxicity.

In this retrospective case series, we describe five patients with pulmonary mucormycosis who showed an inadequate response to posaconazole and then received bronchoscopic clearance followed by local instillation of amphotericin B deoxycholate. The aim was to summarize the clinical context, procedural details, tolerability, imaging evolution, and short-term to long-term follow-up of this adjunctive strategy.

## Materials and methods

### Study design, patient selection, and definitions

This was a single-center retrospective case series. Medical records were reviewed for patients with a documented diagnosis of pulmonary mucormycosis who received posaconazole-based antifungal therapy at the First Affiliated Hospital, Zhejiang University School of Medicine, between May 2019 and December 2022. Patients were included if they subsequently underwent bronchoscopic local instillation of amphotericin B deoxycholate because of an inadequate response to posaconazole.

For this study, inadequate response was defined as at least one of the following during posaconazole-based therapy: (i) clinical deterioration, such as recurrent or persistent fever, worsening cough, sputum production, hemoptysis, or dyspnea; (ii) radiologic progression, including lesion enlargement, new cavitation or abscess formation, new or enlarging nodules, or worsening obstructive pneumonia; or (iii) absence of meaningful radiologic improvement after approximately 6 weeks of posaconazole-based therapy when clinical suspicion of persistent mucormycosis remained. Because this was a retrospective study and source DICOM measurements were not uniformly available, inflammatory markers and percentage lesion-volume changes were not used as mandatory thresholds.

In assessing whether the clinical course represented inadequate antifungal response rather than immune reconstitution inflammatory syndrome (IRIS), the treating team reviewed the temporal relationship between lesion worsening and immune recovery, the presence or absence of persistent fever or respiratory symptoms, serial CT evolution, microbiologic or histopathologic evidence of persistent mucormycosis, posaconazole exposure when available, and bronchoscopic evidence of obstructing fungal or necrotic material. IRIS was considered less likely when radiologic progression was accompanied by persistent symptoms, compatible microbiologic evidence, visible endobronchial obstruction or purulent/necrotic material corresponding to the dominant lesion, and no alternative explanation such as uncontrolled bacterial infection. Local bronchoscopic treatment was therefore reserved for patients in whom the available clinical, imaging, microbiologic, and bronchoscopic findings favored persistent localized infection with impaired airway drainage or drug delivery rather than a purely inflammatory paradoxical reaction.

Patients were excluded if the available records supported another proven invasive mold infection without evidence of mucormycosis, if uncontrolled bacterial infection was considered the main cause of radiologic progression, if pulmonary surgical resection had been performed immediately before the index local instillation, or if another local pulmonary antifungal intervention had been performed before bronchoscopic amphotericin B instillation.

### Data collection

The following information was extracted from the medical records: age, sex, underlying conditions, presenting symptoms, baseline laboratory findings, diagnostic evidence for mucormycosis, chest CT findings and time points, cranial MRI findings when available, posaconazole formulation and dose, posaconazole trough concentration when monitored, prior amphotericin B exposure and toxicity, bronchoscopic findings, local instillation dose and frequency, adverse events, radiologic evolution, follow-up duration, recurrence, need for surgery, and final clinical status.

### Bronchoscopic local instillation protocol

Flexible bronchoscopy was performed when the patient's condition allowed. Chest CT was reviewed before the procedure to identify the affected bronchial segments. Obstructing mucus plugs, purulent secretions, blood clots, or necrotic material were removed as completely as possible by aspiration, lavage, or forceps under direct visualization. Amphotericin B deoxycholate was then instilled slowly into the corresponding segmental or subsegmental bronchi.

The actual dose and schedule varied across cases according to bronchoscopic findings, lesion location, treatment tolerance, and local institutional practice. The documented local doses were 7.5–15 mg amphotericin B deoxycholate diluted in 10–15 ml diluent, administered every 1–2 weeks for 2–3 sessions. Oral or intravenous posaconazole was continued during this period. This regimen was empirical and was not based on an established pulmonary mucormycosis guideline. It was selected according to institutional experience, the need to avoid further systemic amphotericin B toxicity in some patients, and safety extrapolation from reports of non-systemic or directed amphotericin B delivery in invasive fungal disease (19–22).

### Imaging assessment and outcomes

Chest CT scans obtained before and after local treatment were reviewed to assess lesion distribution and interval change. The main radiologic features included nodules, patchy opacities, consolidation, halo sign, reversed halo sign, cavitation, abscess formation, and obstructive pneumonia. Case-level CT time points were extracted and are reported in Table 1. Where the original records described interval change, the description was retained. Formal percentage reduction or volumetric assessment was not performed because retrospective source DICOM datasets and standardized measurement planes were not available for all patients.

**Table 1:** Clinical characteristics, diagnostic evidence, bronchoscopic findings, local instillation regimen, and outcomes of the five included patients.

Case	Sex/age	Underlying condition	Diagnostic evidence	Reason for inadequate response before local therapy	Bronchoscopic findings	Local amphotericin B deoxycholate regimen	CT time points and imaging response	Follow-up/final status
1	Male/57	Diffuse large B-cell lymphoma; prior R-CHOP plus zanubrutinib.	Blood and cerebrospinal fluid mNGS supported pulmonary/CNS mucormycosis; sputum fungal fluorescence showed broad ribbon-like hyphae compatible with mucorales.	Renal dysfunction after systemic amphotericin B cholesterol sulfate complex; CT on 26 Oct 2022 showed left lower-lobe mass-like lesion enlarged vs. 29 Aug 2022 and cavity disappearance despite posaconazole trough 2,381.70 ng/ml.	4 Nov 2022: left lower-lobe dorsal branch mucus plug obstruction, mostly aspirated; 17 Nov 2022: no obvious residual mucus plug.	15 mg in 15 ml diluent on 4 Nov, 17 Nov, and 1 Dec 2022; posaconazole delayed-release tablets 300 mg once daily continued.	29 Aug 2022: improvement; 26/31 Oct 2022: progression; 2 Dec 2022: left lower-lobe lesion smaller than 31 Oct 2022 and some nodules smaller.	No surgical resection; continued posaconazole; stable at latest available follow-up.
2	Female/50	Destroyed lung after pulmonary tuberculosis; previous right upper lobectomy; history of pulmonary aspergillosis.	Bronchoalveolar lavage reported aspergillus plus mucorales in the right upper-lobe bronchial stump lesion.	Persistent cough, sputum, hemoptysis, and no meaningful CT improvement during posaconazole suspension despite trough 2,662.07 ng/ml.	8 Sep 2021: right upper-lobe bronchial stump with mushroom-like fungal material; surrounding mucosa bled easily on contact.	10 mg in 10 ml diluent every 2 weeks for 3 sessions; posaconazole suspension 5 ml four times daily continued.	8 Sep 2021: right upper-lobe destruction, consolidation, calcification, irregular bronchiectasis, cavity formation, and local protruding lesion; interval CT before local therapy showed no meaningful improvement.	No surgical resection; long-term posaconazole; stable at latest available follow-up.
3	Male/55	Acute promyelocytic leukemia after retinoic acid plus arsenic trioxide therapy.	Blood mNGS showed cunninghamella infection; BAL fluorescence staining showed abundant broad, aseptate, branching hyphae compatible with mucorales.	Liver dysfunction after systemic amphotericin B; follow-up CT showed partial absorption of some lesions but progression of a left lower-lung lesion during posaconazole suspension; trough recorded as 2,662.07 ng/ml.	Left lower-lobe superior segment and lateral/posterior branch had thick purulent sputum obstructing the airway.	15 mg in 10 ml diluent once weekly for 3 sessions; posaconazole suspension 5 ml four times daily continued.	CT during posaconazole-based therapy: bilateral infectious lesions with cavitation and left pleural effusion; follow-up CTs in the source record showed absorption/shrinkage of multiple lesions and cavitation in part of the left lung lesions.	No surgical resection; stable at latest available follow-up.
4	Male/59	Small B-cell lymphoma with relapse and bone marrow involvement.	BAL fungal fluorescence showed a small amount of broad branching hyphae compatible with mucorales.	Progression of bilateral pulmonary lesions during hematologic treatment; liver dysfunction after systemic amphotericin B required discontinuation; CT on 27 Aug 2022 showed right upper-lobe mass-like lesion/possible abscess.	Right upper-lobe anterior segment opening was narrowed; mucosa was congested and edematous; purulent secretion overflowed.	10 mg in 10 ml diluent every other week for 2 sessions; posaconazole delayed-release tablets continued.	27 Aug 2022: right upper-lobe mass-like lesion/possible abscess; 6 Sep 2022: partial absorption; 8 Dec 2022: further absorption of right upper-lobe and scattered bilateral lesions.	No surgical resection; stable and able to continue treatment for hematologic disease.
5	Female/60	History of pulmonary mucormycosis after lung surgery; later sinonasal fungal disease.	Previous lung surgical pathology suggested mucormycosis; 2018 lung nodule biopsy pathology showed mucorales; later sinonasal pathology showed aspergillus.	During posaconazole-based therapy, CT on 20 May 2019 showed multiple bilateral masses/nodules, with some lesions enlarged vs. 24 April 2019.	29 May 2019: left upper-lobe post-operative stump; right upper-lobe posterior segment sputum obstruction; lumen slightly narrowed after clearance.	7.5 mg in 10 ml diluent on 29 May and 12 Jun 2019; posaconazole suspension 5 ml four times daily continued.	24 Apr 2019: pulmonary nodules enlarged; 20 May 2019: some masses/nodules enlarged; 6 Nov 2019: multiple nodules partly smaller and partly unchanged; 25 July 2022: multiple nodules smaller.	No surgical resection after local therapy; cough and sputum improved; stable at latest available follow-up.

BAL, bronchoalveolar lavage; CNS, central nervous system; CT, computed tomography; mNGS, metagenomic next-generation sequencing.

The primary descriptive outcomes were procedural tolerability, short-term adverse events, radiologic response, need for subsequent surgical resection, recurrence, survival status, and final clinical stability during follow-up.

### Statistical analysis

Because of the small sample size, only descriptive statistics were used. Continuous variables are presented as mean ± standard deviation, and categorical variables are summarized as counts and percentages.

### Ethics statement

The study was approved by the Institutional Review Board of Clinical Research of the First Affiliated Hospital, Zhejiang University School of Medicine (IIT-2023–0144). Written informed consent for participation and publication of anonymized clinical information and imaging data was obtained from all patients.

## Results

### Patient characteristics and diagnostic evidence

Nineteen patients with pulmonary mucormycosis received posaconazole-based antifungal therapy during the study period. Five patients met the eligibility criteria after showing an inadequate response to posaconazole and subsequently receiving bronchoscopic clearance plus local instillation of amphotericin B deoxycholate. Three patients were men and two were women, and the mean age was 56.2 ± 3.96 years. Four patients had major underlying conditions: three had hematologic malignancies and one had a destroyed lung after pulmonary tuberculosis. The remaining patient had a history of pulmonary mucormycosis after lung surgery and later sinonasal fungal disease.

Case-level diagnostic evidence is summarized in Table 1 to avoid duplicating the table in the Results. In brief, the diagnosis of pulmonary mucormycosis was supported by metagenomic next-generation sequencing, fungal fluorescence staining of sputum or bronchoalveolar lavage specimens, bronchoalveolar lavage findings, and/or histopathology, depending on the clinical context of each patient.

### Baseline laboratory and imaging findings

At the time pulmonary mucormycosis was diagnosed, all five patients underwent routine blood testing, procalcitonin measurement, and high-sensitivity C-reactive protein testing. Mean values were as follows: white blood cell count, 6.03 ± 3.61 x 10^9^/L; absolute neutrophil count, 4.23 ± 2.91 x 10^9^/L; absolute lymphocyte count, 1.23 ± 0.60 x 10^9^/L; C-reactive protein, 48.09 ± 28.92 mg/L; and procalcitonin, 0.11 ± 0.09 ng/ml. beta-1,3-D-glucan was < 10 pg/ml in all cases. Galactomannan was low in the cases with available data.

### Radiologic evolution and treatment history before local therapy

In three patients, pulmonary lesions initially improved during treatment that included amphotericin B and posaconazole, but later progressed after amphotericin B had been discontinued because of hepatic or renal dysfunction and posaconazole was continued alone. In the remaining two patients, lesions failed to improve meaningfully during posaconazole-based therapy. Posaconazole delayed-release tablets were used in Cases 1 and 4, whereas posaconazole suspension was used in Cases 2, 3, and 5; Case 5 also received intravenous posaconazole during hospitalization. Posaconazole trough concentrations were available in three documented measurements: 2,381.70 ng/ml in Case 1 and 2,662.07 ng/ml in Cases 2 and 3. These values suggested that inadequate response was unlikely to be explained only by systemic underexposure in those cases.

### Bronchoscopic findings, local treatment, and safety

Bronchoscopy showed obstructing mucus plugs, purulent secretions, necrotic material, bronchial stump fungal material, or segmental narrowing in all five patients. The involved sites corresponded to the dominant radiologic lesions. After endobronchial clearance, amphotericin B deoxycholate was instilled locally. Case 1 received 15 mg in 15 ml diluent on three occasions; Case 2 received 10 mg in 10 ml diluent every 2 weeks for three sessions; Case 3 received 15 mg in 10 ml diluent weekly for three sessions; Case 4 received 10 mg in 10 ml diluent every other week for two sessions; and Case 5 received 7.5 mg in 10 ml diluent on two occasions. Posaconazole was continued during and after local therapy.

All five patients tolerated the procedure. The available records did not document procedure-related fever, treatment-limiting cough, clinically significant oxygen desaturation, hemoptysis, wheezing, bronchospasm, or new hepatic or renal dysfunction attributable to the local instillation. No patient required emergency airway intervention after the procedure.

### Radiologic response and follow-up

Follow-up chest CT showed partial absorption or shrinkage of pulmonary lesions in all five patients. Case 1 showed shrinkage of the left lower-lobe lesion on 2 December 2022 compared with CT before local treatment. Case 2 remained clinically stable after local therapy and continued posaconazole. Case 3 showed absorption or shrinkage of multiple lesions on follow-up CT. Case 4 showed partial absorption of the right upper-lobe lesion on 6 September 2022 and further absorption on 8 December 2022. Case 5 showed partial reduction or stability of multiple nodules on 6 November 2019 and further shrinkage on 25 July 2022. None of the patients required surgical resection after local instillation, and all were described as clinically stable at the latest available follow-up.

**Figure 1:**
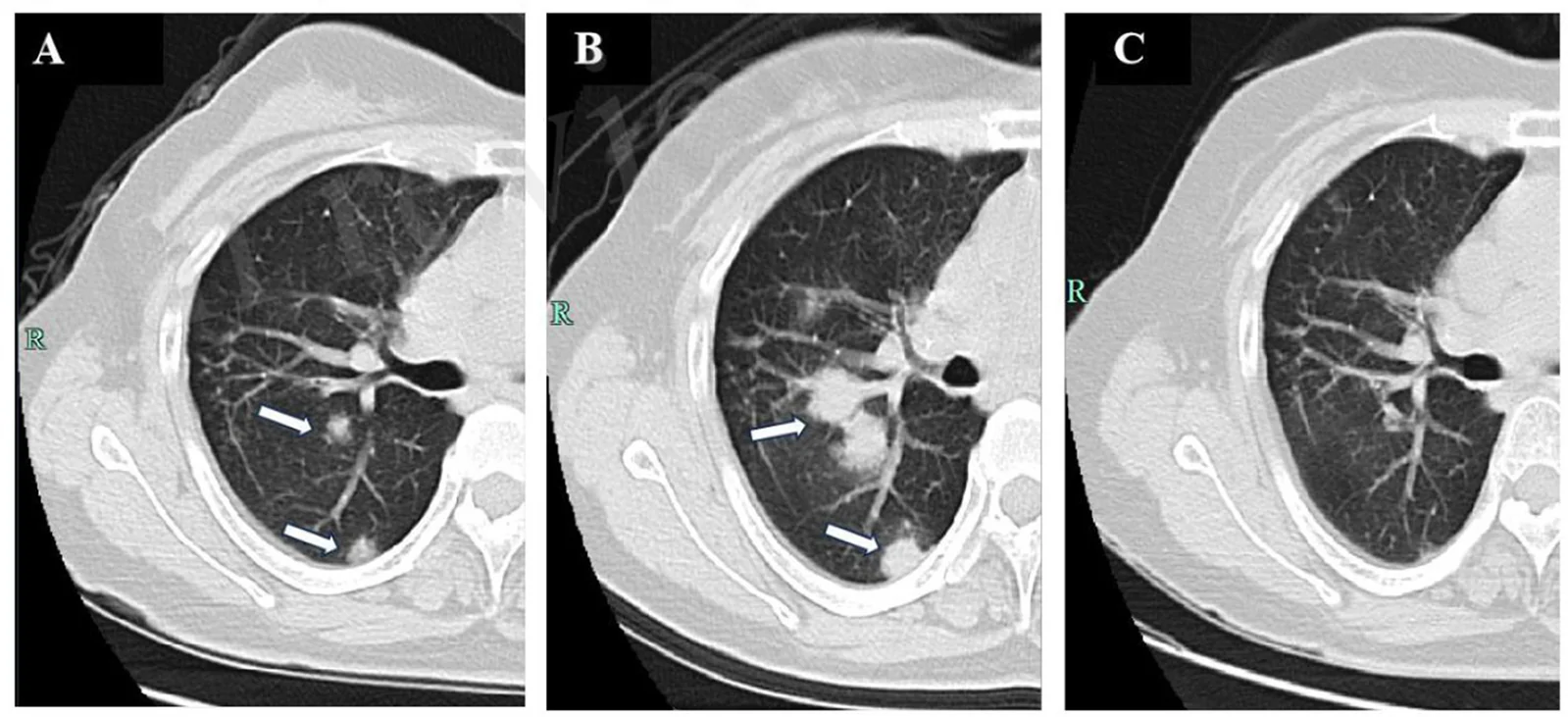
Serial chest CT images of Case 1. Panels A-C show the baseline examination during posaconazole treatment (29 August 2022), interval radiologic progression before local therapy (31 October 2022), and lesion improvement after bronchoscopic airway clearance plus local instillation of amphotericin B deoxycholate (2 December 2022), respectively. Arrows indicate the dominant lesions of interest. The images have been de-identified.

Representative serial CT images from Case 1 are shown in Figure 1. Additional representative CT and bronchoscopic images from Cases 2–5 are provided in Supplementary Figure S1

## Discussion

This case series suggests that bronchoscopic clearance combined with local instillation of amphotericin B deoxycholate may be a feasible adjunct in selected patients with pulmonary mucormycosis who do not respond adequately to posaconazole. The key clinical context was not simply persistent infection, but persistent infection in the presence of visible endobronchial obstruction, purulent secretions, bronchial stump fungal material, or segmental narrowing. In that setting, continued systemic therapy alone may not provide sufficient drug exposure or drainage at the diseased site, even when plasma posaconazole concentrations are within commonly used therapeutic ranges.

Clinically, IRIS and inadequate antifungal response can both present with worsening symptoms or enlarging lesions. In this series, the decision to perform bronchoscopic clearance and local amphotericin B instillation was not based on imaging enlargement alone. The key factors supporting inadequate local therapeutic effect were persistent or recurrent respiratory manifestations, radiologic progression or lack of meaningful absorption during posaconazole-based therapy, compatible diagnostic evidence for mucormycosis, and direct bronchoscopic visualization of mucus plugs, purulent secretions, necrotic material, bronchial stump fungal material, or segmental narrowing at the involved sites. Conversely, cases in which deterioration was more plausibly explained by isolated inflammatory rebound during immune recovery, without endobronchial obstruction or evidence of persistent fungal burden, would not be considered appropriate candidates for local antifungal instillation under this protocol.

Pulmonary mucormycosis remains difficult to treat because the disease combines rapid angioinvasion, tissue infarction, and host immunosuppression (4, 5, 10–15, 17). Current guidelines recommend liposomal amphotericin B as the preferred initial therapy, with posaconazole and isavuconazole used as salvage, step-down, or alternative options (15, 16, 18). In everyday practice, however, toxicity often limits prolonged use of amphotericin B formulations. In our series, several patients developed hepatic or renal dysfunction during systemic amphotericin B treatment, and treatment had to be de-escalated to posaconazole. This is consistent with the well-recognized toxicity profile of amphotericin B deoxycholate and explains why alternative delivery strategies remain clinically relevant (23–27). Posaconazole exposure and clinical effectiveness may vary according to formulation, pharmacokinetic factors, and patient context, supporting therapeutic drug monitoring and individualized assessment when response is suboptimal (28–31). Across invasive mold disease, published strategies include posaconazole prophylaxis or salvage treatment, as well as high-dose and combination antifungal approaches for difficult-to-treat infections, including mucormycosis (32–35).

Several non-systemic approaches have been explored to reduce systemic exposure to amphotericin B, including aerosolized inhalation and local irrigation or directed injection in invasive fungal disease (19–22). For pulmonary mucormycosis with obstructed bronchi, inhaled therapy may not adequately reach the diseased segments. Bronchoscopy offers two potential advantages in this setting. First, it allows direct removal of endobronchial secretions, blood clots, and necrotic material. Second, it allows the antifungal agent to be delivered directly to the involved bronchial tree under visual guidance. In our patients, these two steps were performed together.

The most important alternative explanation for the observed improvement is mechanical airway clearance. Removing mucus plugs, purulent secretions, or necrotic debris can itself improve post-obstructive pneumonia, ventilation, drainage, and radiologic appearance. Because clearance and amphotericin B instillation were performed in the same procedure, this case series cannot isolate the incremental benefit of local amphotericin B. Therefore, the intervention should be interpreted as a combined bronchoscopic salvage approach rather than proof that local amphotericin B alone was effective.

The local dosing strategy also requires caution. The original clinical protocol was not standardized: the administered dose ranged from 7.5 to 15 mg, the volume from 10 to 15 ml, the interval from 1 to 2 weeks, and the number of sessions from 2 to 3. These choices were empirical and reflected lesion extent, airway tolerance, and clinician judgment. Future studies should define the optimal formulation, diluent, dose, dwell time, interval, and total number of sessions, and should evaluate whether local drug exposure can be measured pharmacokinetically.

The study has several additional limitations. It was retrospective, single-center, uncontrolled, and included only five patients. Posaconazole formulations varied, and therapeutic drug monitoring was not uniformly performed. Imaging response was assessed retrospectively, and formal volumetric or RECIST-like measurement was not possible for all cases because original DICOM measurements were not uniformly available. Diagnostic evidence also differed across patients, reflecting real-world practice in a rare disease. Mechanical clearance, continued systemic posaconazole, host immune recovery, treatment of the underlying disease, and the natural disease course may all have contributed to the observed improvement.

Despite these limitations, the present series has practical value because it addresses a specific salvage scenario: pulmonary mucormycosis with inadequate response to systemic therapy and visible endobronchial obstruction. For clinicians managing similar patients, bronchoscopic clearance followed by carefully selected local amphotericin B deoxycholate instillation may be considered as an adjunctive option when bronchoscopy is feasible and when additional systemic amphotericin B exposure is undesirable. Prospective multicenter studies are needed to define patient selection, safety, optimal dose and interval, standardized imaging endpoints, and the true incremental benefit of local antifungal delivery.

## Conclusion

Adjunctive bronchoscopic clearance plus local instillation of amphotericin B deoxycholate appeared feasible and well tolerated in this small series of patients with pulmonary mucormycosis and inadequate response to posaconazole. The approach may be most relevant when endobronchial obstruction is thought to impair local drainage and drug delivery. However, the effects of mechanical clearance and local amphotericin B delivery cannot be separated in this retrospective study, and efficacy should be confirmed in larger prospective studies.

## Data Availability

The raw data supporting the conclusions of this article will be made available by the authors, without undue reservation.

## Supplementary material

Supplementary Figure 1 Representative de-identified serial CT and bronchoscopic images from Cases 2–5. Titles are provided for each panel to improve readability: Case 2, right upper-lobe destroyed lung and bronchial stump fungal material; Case 3, serial left lung lesions with bronchoscopic airway obstruction; Case 4, right upper-lobe mass-like lesion/possible abscess with interval partial absorption and bronchoscopic purulent secretion; and Case 5, bilateral pulmonary nodules before and after local therapy with representative bronchoscopic findings. **(A)** Case 2: serial CT images showing right upper-lobe destroyed lung and interval follow-up before/after local therapy. **(B)** Case 2: bronchoscopic view of fungal material at the right upper-lobe bronchial stump. **(C)** Case 3: serial CT images of left lung lesions during posaconazole-based therapy and follow-up. **(D)** Case 3: follow-up CT image after bronchoscopic local treatment. **(E)** Case 4: right upper-lobe mass-like lesion/possible abscess before local therapy. **(F)** Case 4: follow-up CT showing interval partial absorption after local therapy. **(G)** Case 5: bilateral pulmonary nodules/masses before local bronchoscopic therapy. **(H)** Case 5: follow-up CT showing lesion reduction or stability after local therapy.
