# Supplementary material for: Bronchoscopic local instillation of amphotericin B deoxycholate as adjunctive therapy for pulmonary mucormycosis with inadequate response to posaconazole: a retrospective case series
Source: Front Med (Lausanne). 2026 Aug 17;13:1840224. doi: 10.3389/fmed.2026.1840224 (PMC13526596; doi:10.3389/fmed.2026.1840224)

Figure 1. Serial chest CT images of Case 1.

Panels A-C show the baseline examination during posaconazole treatment, interval radiologic progression before local therapy, and lesion improvement after bronchoscopic local instillation of amphotericin B deoxycholate, respectively.

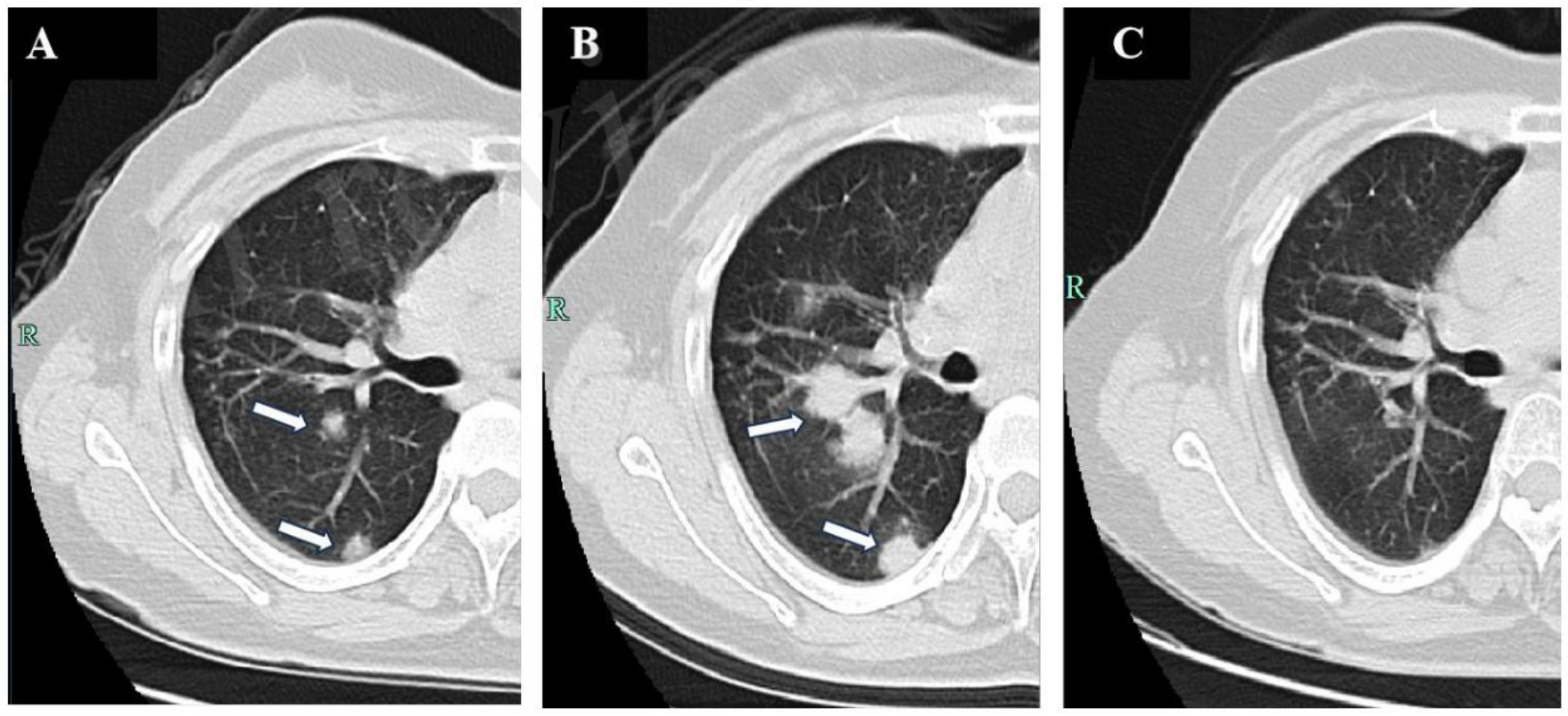

Supplementary Figure 1. Representative de-identified serial CT and bronchoscopic images from Cases 2-5. Titles are provided for each panel to improve readability: Case 2, right upper-lobe destroyed lung and bronchial stump fungal material; Case 3, serial left lung lesions with bronchoscopic airway obstruction; Case 4, right upper-lobe mass-like lesion/possible abscess with interval partial absorption and bronchoscopic purulent secretion; and Case 5, bilateral pulmonary nodules before and after local therapy with representative bronchoscopic findings.

Supplementary Figure 1A. Case 2: serial CT images showing right upper-lobe destroyed lung and interval follow-up before/after local therapy.

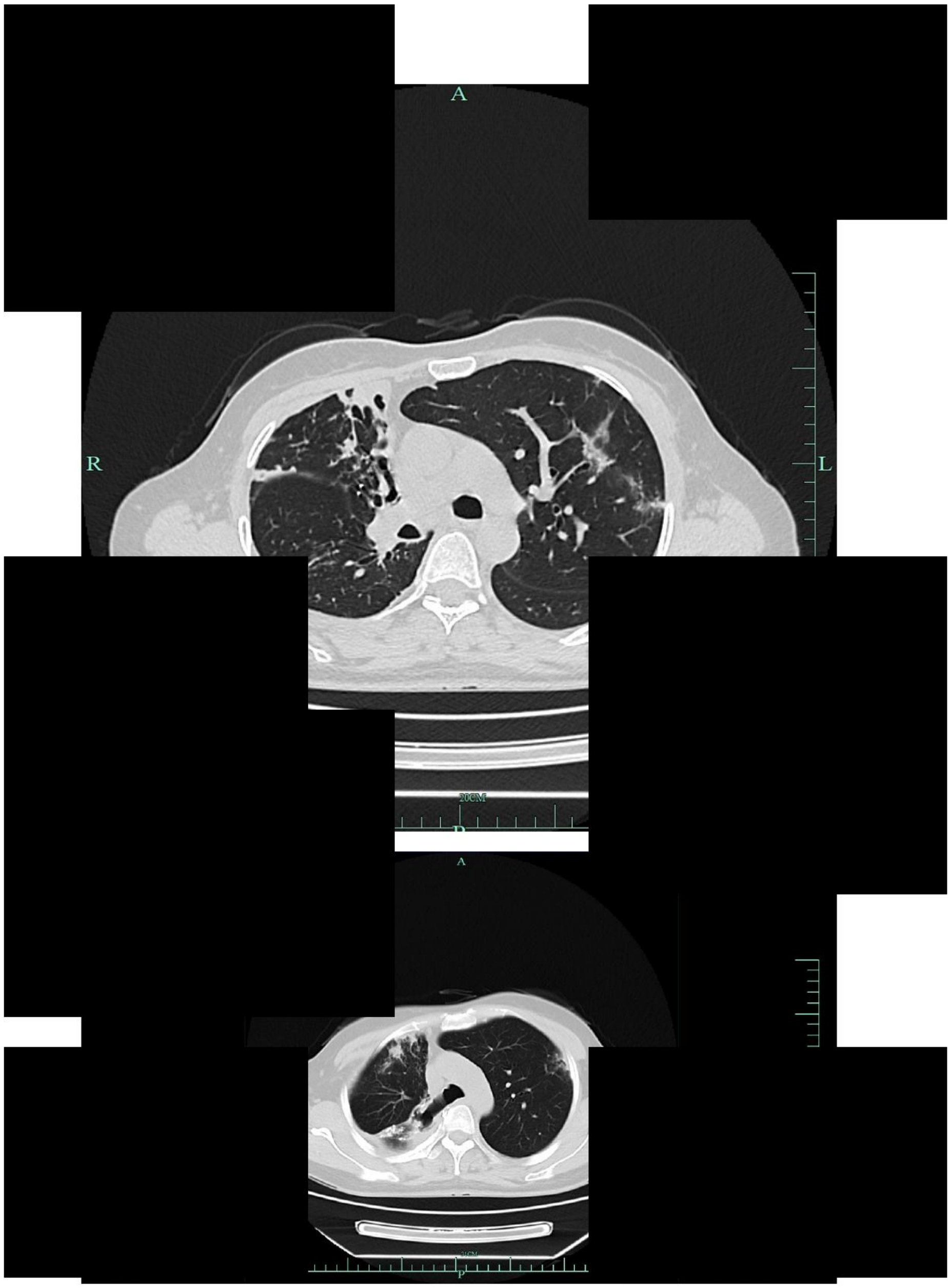

Supplementary Figure 1B. Case 2: bronchoscopic view of fungal material at the right upper-lobe bronchial stump.

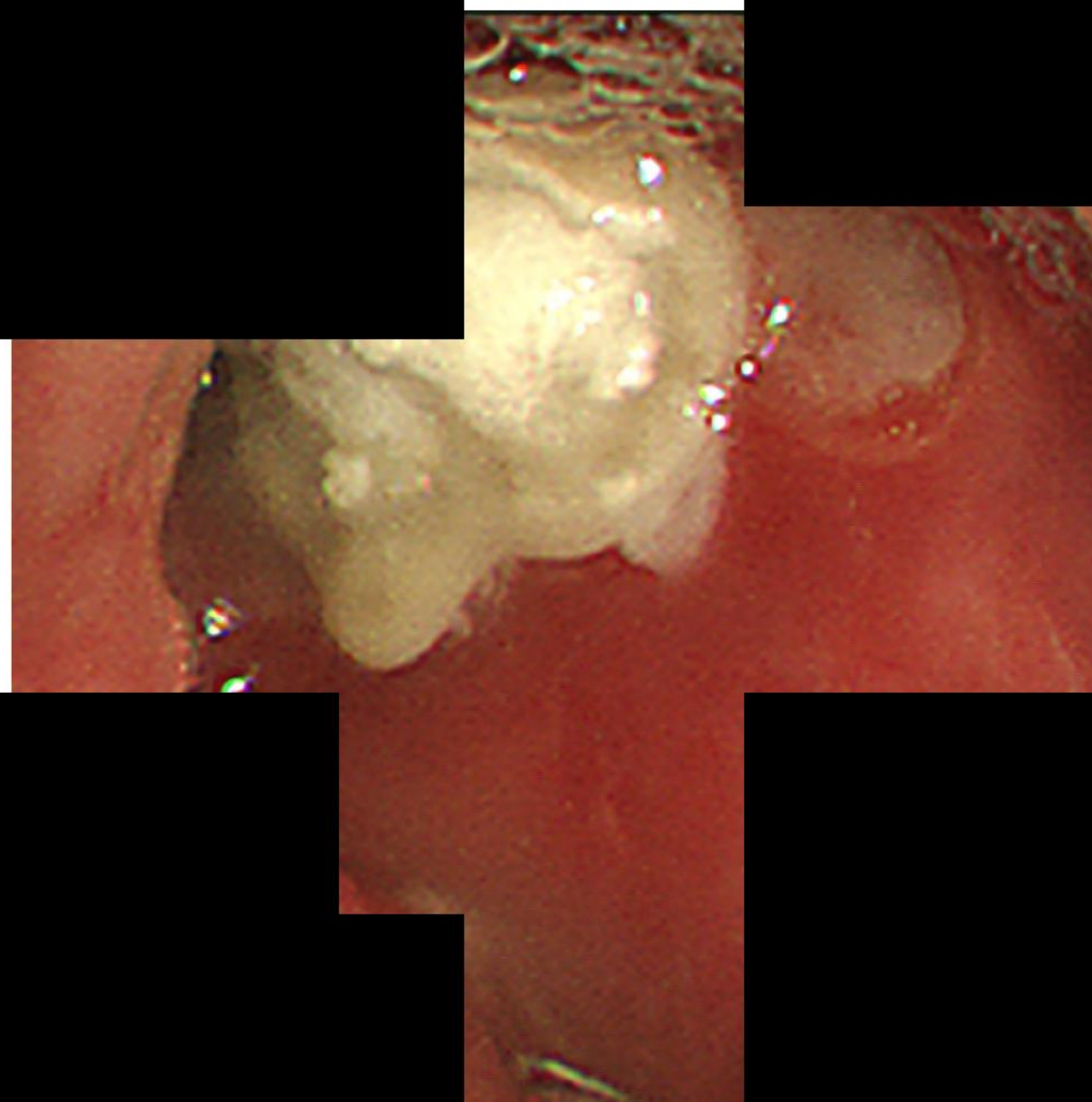

[redacted]

Supplementary Figure 1C. Case 3: serial CT images of left lung lesions during posaconazole-based therapy and follow-up.

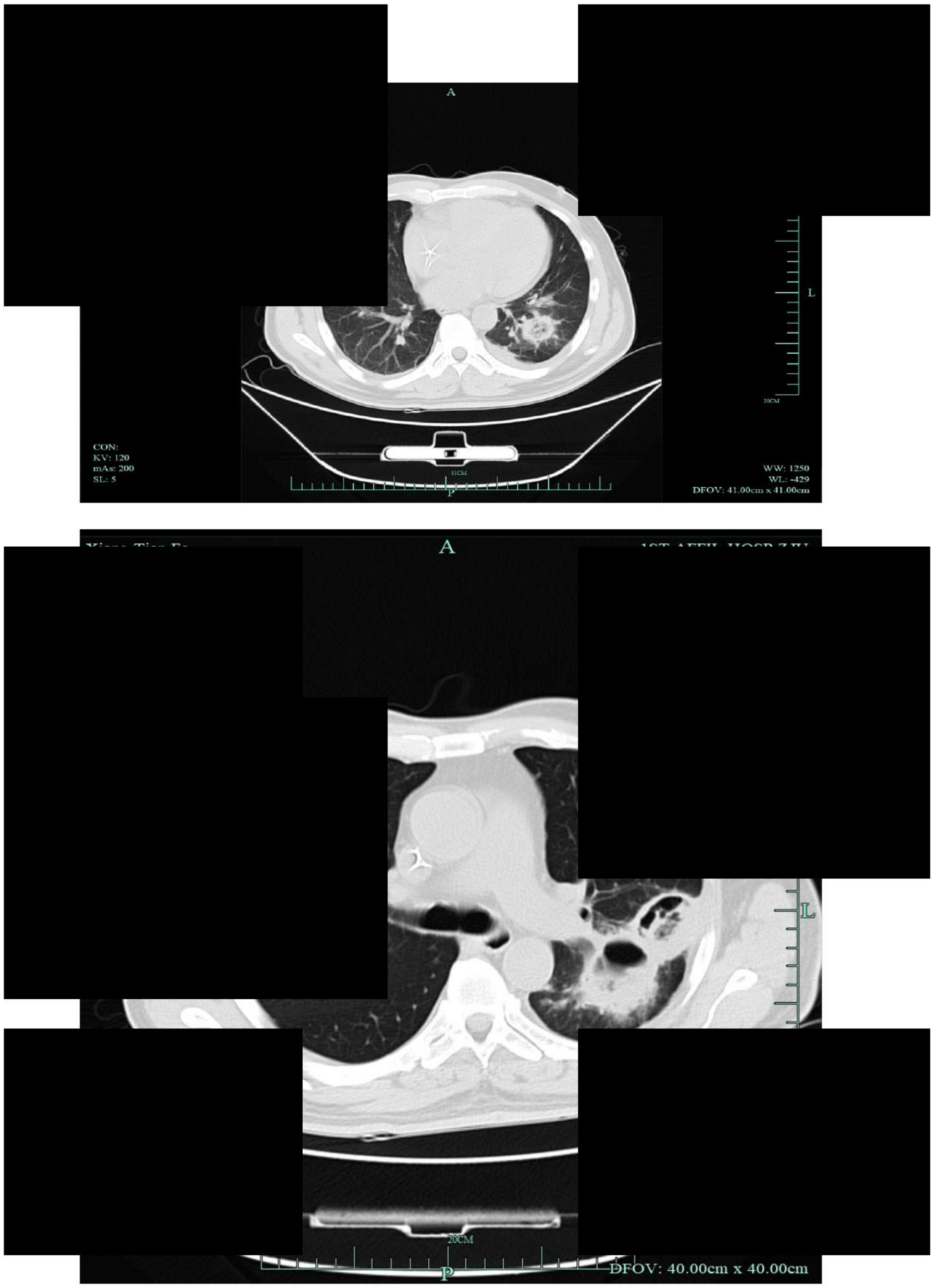

Supplementary Figure 1D. Case 3: follow-up CT image after bronchoscopic local treatment.

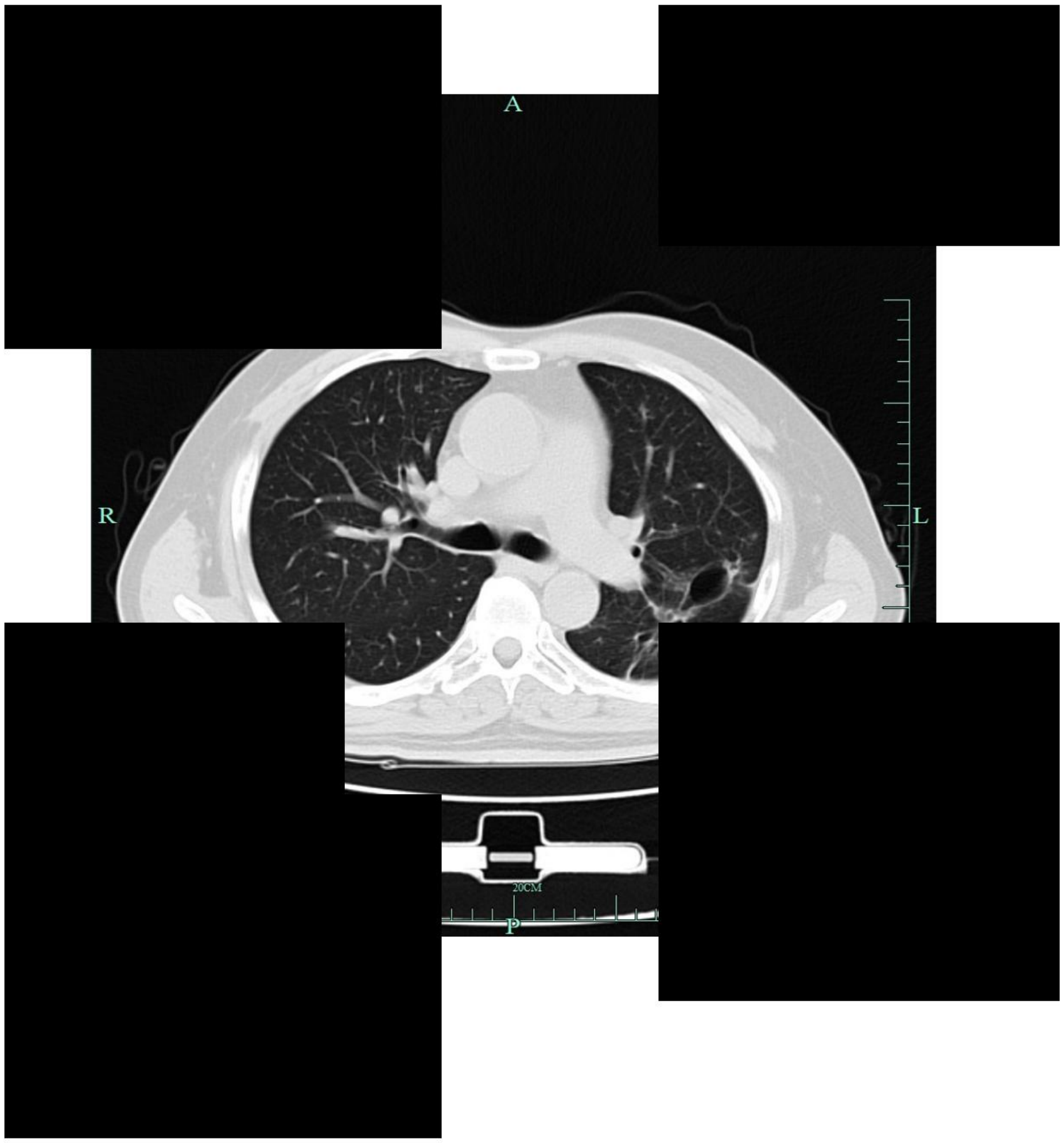

[redacted]

Supplementary Figure 1E. Case 4: right upper-lobe mass-like lesion/possible abscess before local therapy.

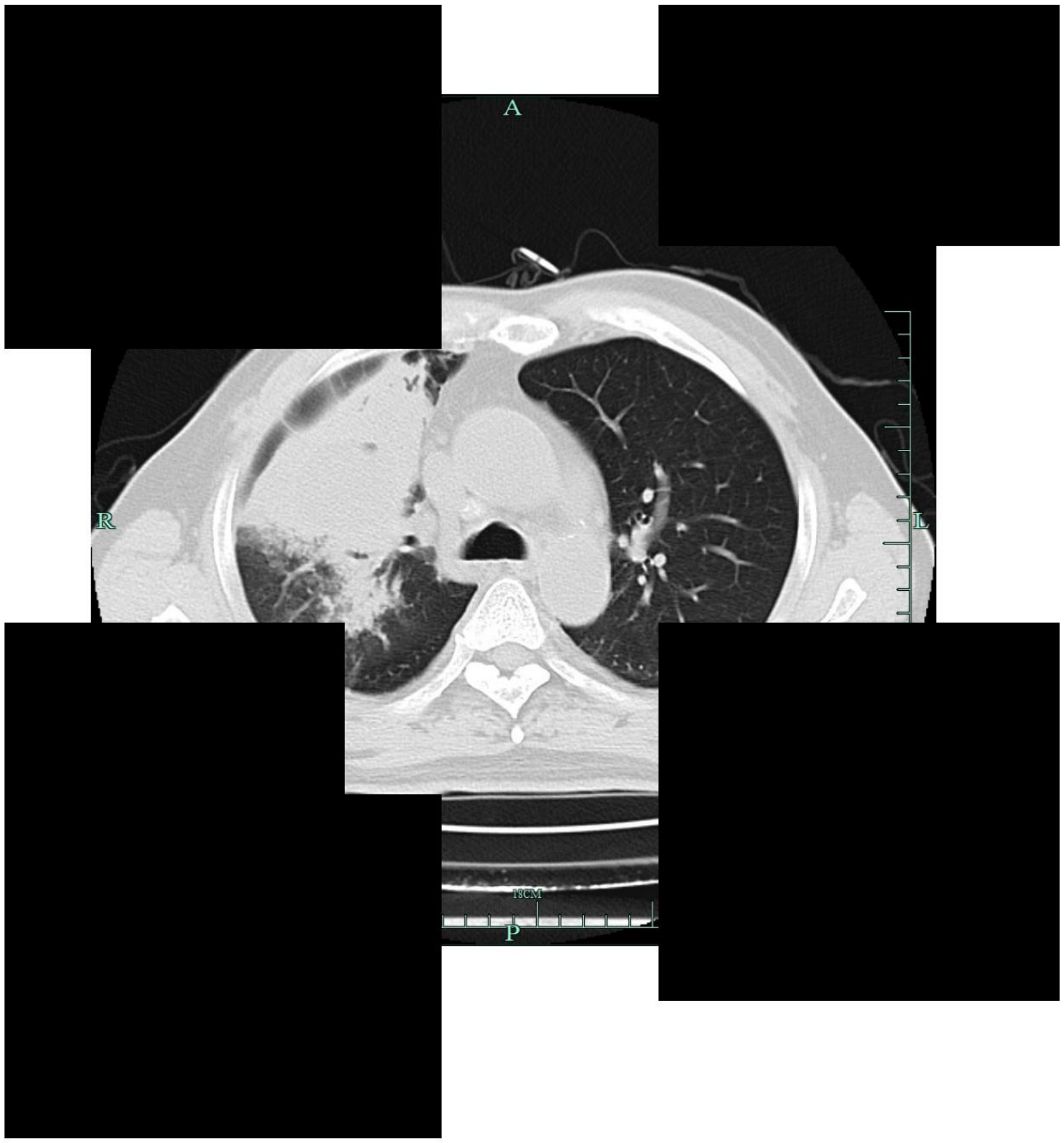

[redacted]

Supplementary Figure 1F. Case 4: follow-up CT showing interval partial absorption after local therapy.

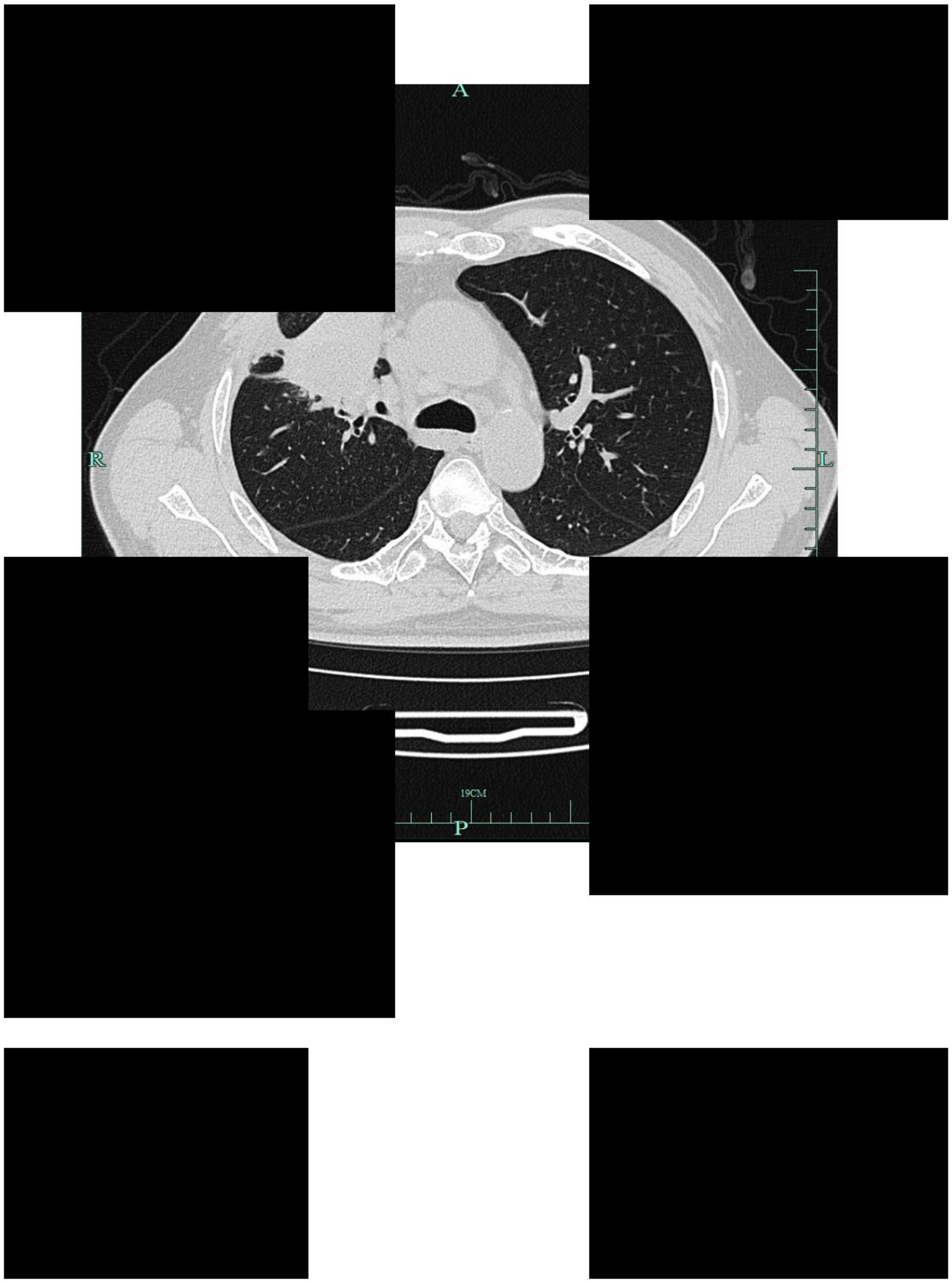

Supplementary Figure 1G. Case 5: bilateral pulmonary nodules/masses before local bronchoscopic therapy.

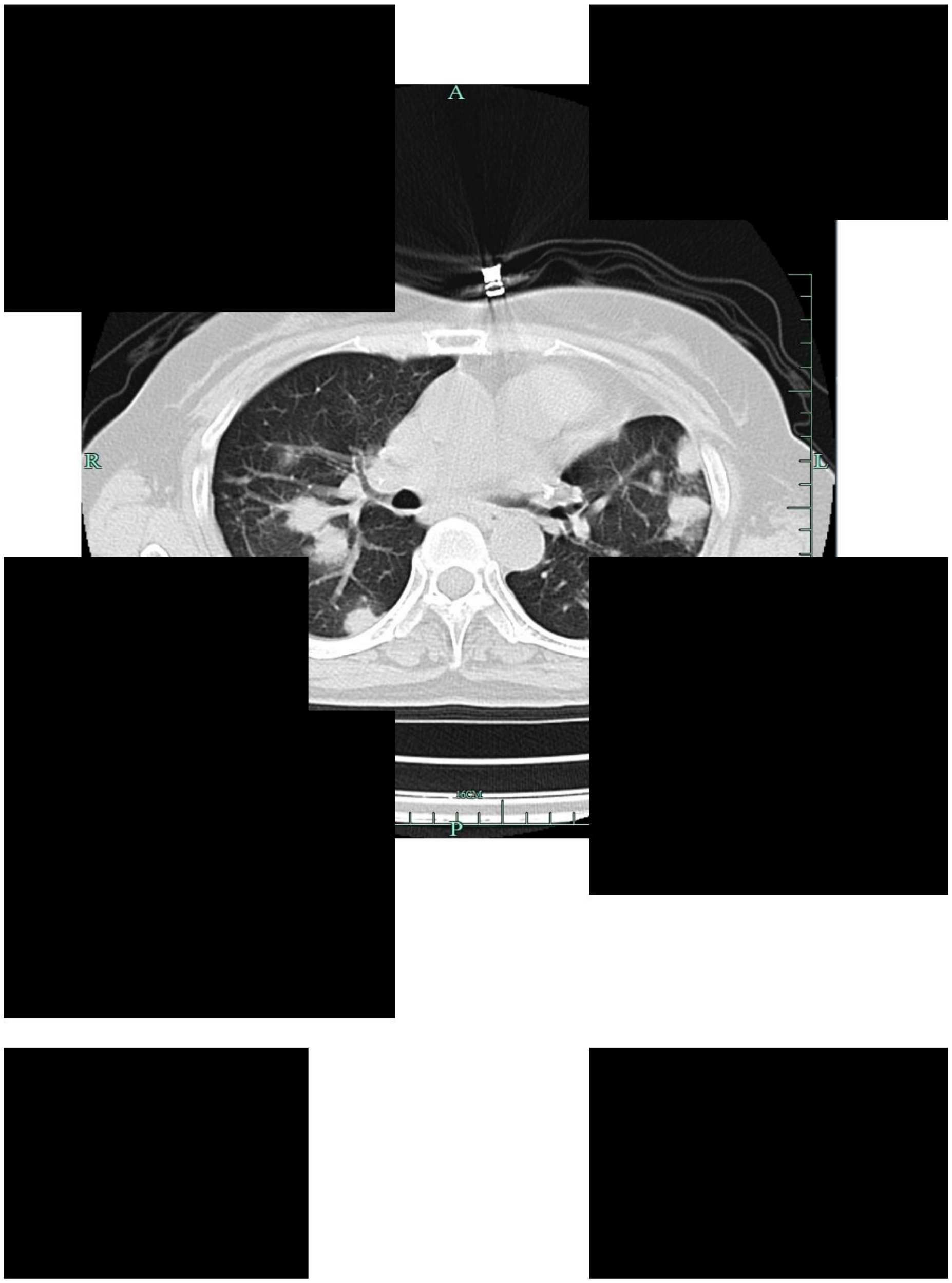

Supplementary Figure 1H. Case 5: follow-up CT showing lesion reduction or stability after local therapy.

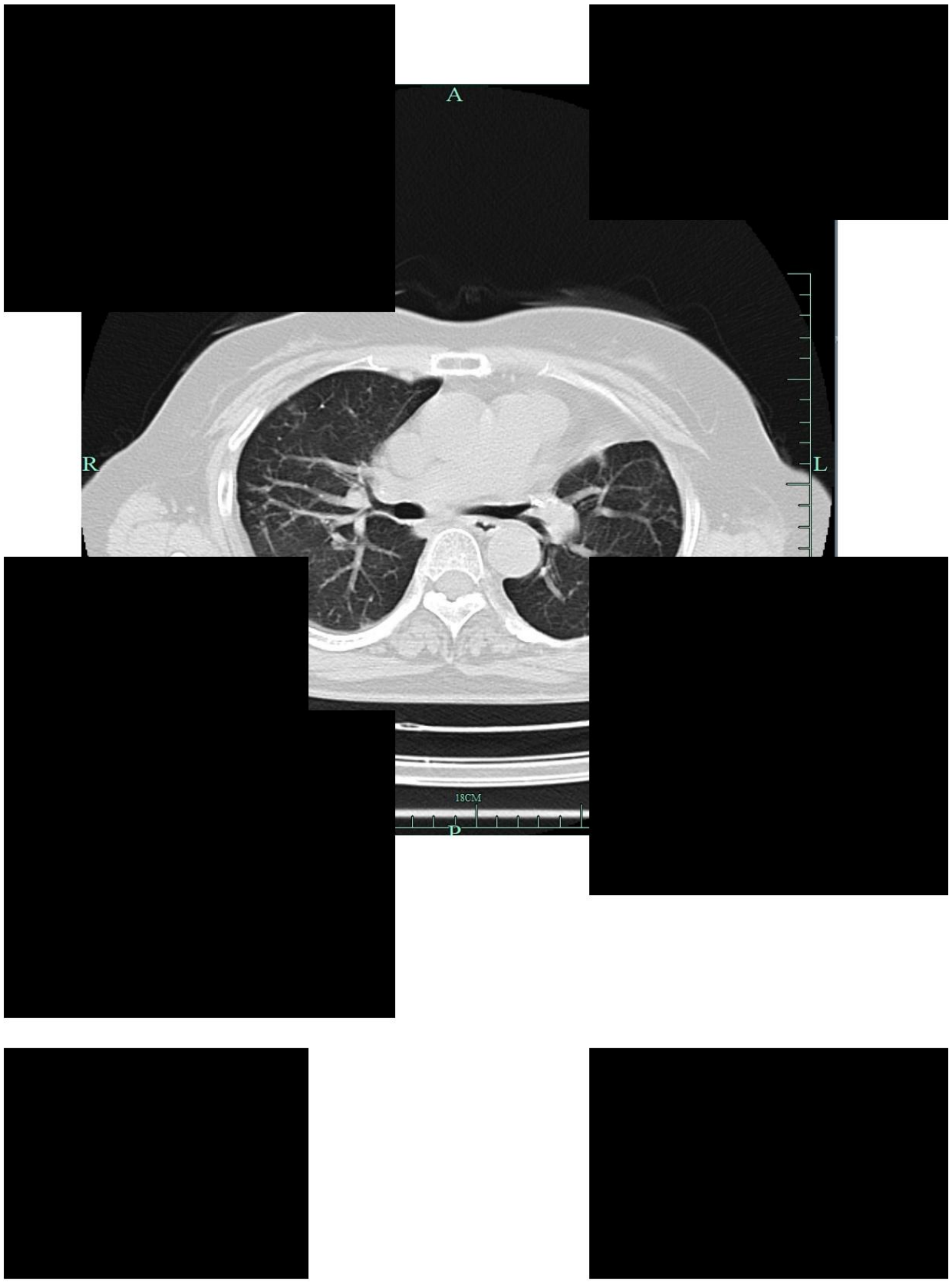

Supplement: Supplementary file 1 [file Supplementary_file_1.pdf]
